# Baseline Albumin-Bilirubin grade as a predictor of response and outcome of regorafenib therapy in patients with hepatocellular carcinoma: a systematic review and meta-analysis

**DOI:** 10.1186/s12885-023-11488-9

**Published:** 2023-10-19

**Authors:** Huilin Xu, Dedong Cao, Dingjie Zhou, Nan Zhao, Xixian Tang, Vishalkumar G. Shelat, Hrishikesh Samant, Sanjaya K. Satapathy, Francisco Tustumi, Giuseppe Aprile, Anbing He, Ximing Xu, Wei Ge

**Affiliations:** 1 Department of Oncology, The Fifth Hospital of Wuhan, #122 Xianzheng Road, Hanyang District, Wuhan, 430000 China; 2 Department of Oncology, Renmin Hospital of Wuhan University, Wuhan, China; 3 Department of General Surgery, Tan Tock Seng Hospital, Singapore, Singapore; 4 Division of Hepatology, Ochsner Multi-Organ Transplant Center, New Orleans, LA USA; 5 Division of Hepatology, Department of Medicine and Northwell Center for Liver Diseases & Transplantation, Northwell Health, Manhasset, NY USA; 6 Department of Gastroenterology, Digestive Surgery Division, University of São Paulo Medical School, Sao Paulo, Brazil; 7 Department of Oncology, San Bortolo General Hospital, Berica, Vicenza, Italy; 8 Department of Oncology, Taikang Tongji Hospital of Wuhan, Wuhan, China

**Keywords:** Hepatocellular carcinoma (HCC), Regorafenib, Biomarker, Albumin-bilirubin (ALBI), Survival

## Abstract

**Background:**

The use of regorafenib in the treatment of hepatocellular carcinoma (HCC) is widespread. Albumin-Bilirubin (ALBI) has been shown to be a potential prognostic marker for regorafenib treatment, but its prognostic value remains controversial. Therefore, we conducted a meta-analysis to investigate the value of the baseline ALBI grade in predicting the efficacy and survival outcomes of HCC patients after regorafenib treatment.

**Methods:**

PubMed, Embase, Cochrane library, Web of Science, CNKI, Wan Fang Data, and Vip Database were searched from January 2010 to October 2022. Studies treating HCC patients with regorafenib and with ALBI as a categorical variable, overall survival (OS) and progression-free survival (PFS) as outcome indicators were included. After applying Newcastle–Ottawa Scale (NOS) to evaluate the quality of the included studies, Review Manager 5.4 was used to statistically analyze. Chi-square Q test and I^2^ statistics were used to detect heterogeneity. Funnel plot asymmetry, Egger’s and Begg’s test were used to evaluate publication bias.

**Results:**

A total of 12 studies, comprising 1,918 patients, were included in the meta-analysis. The included studies were all evaluated as high quality. Compared to the high-grade baseline ALBI group, patients in the low-grade group had a longer survival time after receiving regorafenib and also more suitable for regorafenib treatment [odds ratio (OR) = 6.50, 95% confidence interval (CI): 2.22–18.96, *P* < 0.01]. The low-grade baseline ALBI group before sorafenib treatment was significantly correlated with better OS [hazard ratio (HR) = 2.36, 95% CI: 1.68–3.31, *P* < 0.00001] and PFS (HR = 1.56, 95% CI: 1.16–2.08, *P* = 0.003). Likewise, the low-grade baseline ALBI group before regorafenib was also significantly correlated with better OS (HR = 1.56, 95% CI: 1.15–2.13, *P* = 0.005) and PFS (HR = 2.06, 95% CI: 1.37–3.11, *P* = 0.0005). In addition, the ALBI grade was significantly correlated with disease control rate (DCR) (OR = 2.90, 95% CI: 1.45–5.79, *P* = 0.003), but not the objective response rate (OR = 1.98, 95% CI: 0.71–5.46, *P* = 0.19).

**Conclusions:**

The baseline ALBI grade could be a valuable prognostic indicator for predicting response and outcomes in HCC patients treated with regorafenib.

**Supplementary Information:**

The online version contains supplementary material available at 10.1186/s12885-023-11488-9.

## Introduction

Liver cancer ranks as the 6^th^ in incidence and 3^rd^ in termsof mortality [1, 2]. Among liver cancers, hepatocellular carcinoma (HCC) is the most common malignancy [3]. Several high-risk factors can lead to the occurrence of liver cancer, including hepatitis B virus, hepatitis C virus, aflatoxin, alcohol, fatty liver disease, and other conditions [1, 4]. Currently, there are various treatments for HCC, including surgical treatment, local ablation, transarterial chemoembolization (TACE), radiotherapy, immunotherapy, targeted therapy, and chemotherapy [5–7]. The 5-year survival rate of HCC patients who are candidates for curative therapies has been reported to exceed 70% [8]. However, with a median survival time of 6 to 12 months, the prognosis of advanced HCC patients is usually poor [9]. Dramatic progress in the treatment landscape has been made. However, the treatment strategies have certain liver function requirements [10]. Thus, it is necessary to establish a simple and effective way of assessing liver function to facilitate treatment and predict the clinical efficacy and prognosis of these patients.

Currently, the Child–Pugh score is the most commonly used criteria to evaluate liver function and has been incorporated into the Barcelona Clinic Liver Cancer (BCLC) staging system to guide the clinical treatment and prognosis of HCC patients [11–14]. However, 2 (i.e., ascites and hepatic encephalopathy) of the 5 indicators, which also include albumin, total bilirubin, and prothrombin time, are highly subjective. In addition, albumin and ascites are also commonly affected by other factors, which reduce the objectivity and accuracy of the scoring system [15, 16]. The Albumin-Bilirubin (ALBI) grade is a new classification method proposed in recent years to evaluate liver function, which only contains two objective indicators (i.e., albumin and bilirubin) [17]. Previous reports showed that HCC patients with normal liver function have a low ALBI grade, which usually impacts survival rates [18].

For a long period, sorafenib, a multi-target tyrosine kinase inhibitor, has been recommended as a first-line treatment regimen for advanced HCC patients [19, 20]. This is exciting time in the field of systemic chemotherapy by introduction of immune checkpoint inhibitors. If first line treatment regimen fails, regorafenib is suggested as the subsequent-line therapy for the treatment of HCC, and it has been shown to have considerable efficacy [19, 21–25]. It has been shown that TACE combined with regorafenib and PD-1 antibody has a higher DCR after failure of TACE combined with regorafenib second-line therapy [26]. Studies have shown that the ALBI grade can be used to predict the prognosis of HCC patients treated with regorafenib [27–29]. An analysis of 284 regorafenib-treated HCC patients showed that the ALBI grade was an independent predictor of overall survival (OS), and that ALBI grade 1 patients had a 32% lower risk of death than ALBI grade 2 patients [hazard ratio (HR) = 0.68, *P*= 0.08] [30]. However, other study showed no significant correlation between ALBI grade and OS [31]. The value of the ALBI grade in the prognostic assessment of HCC patients treated with regorafenib remains controversial, and most of the original studies did not address this issue.

Thus, systematic review and meta-analysis are needed to explore the predictive value of the baseline ALBI grade in the long-term prognosis of HCC patients who had been treated with regorafenib.

## Methods

### Systematic search

Public online databases, such as PubMed, Embase, Cochrane Library, Web of Science, CNKI, Wan Fang Data, and Vip Database were searched to retrieve potential eligible studies. The English search terms were as follows: (hepatocellular carcinoma or HCC or liver cell carcinoma) and (ALBI or Albumin-Bilirubin grade) and (regorafenib). The search was for the period from January 2010 until October 2022. To ensure all eligible studies possible were included, the reference lists of the included studies were also screened, and any relevant articles identified were manually retrieved.

### Inclusion and exclusion criteria

Inclusion criteria were based on PICOS principles: (I) patients: include HCC patients whose diagnosis had been confirmed by pathology and/or imaging; (II) Interventions: patients with HCC received regorafenib. (III) Comparisons: ALBI values that were presented as categorical variables and the survival benefit of these patients was compared according to ALBI levels. (IV) Outcomes: use OS and progression-free survival (PFS) as the outcome indicators. (V) have obtainable effect estimates [e.g., the HR, odds ratio (OR) and its 95% confidence interval (CI)]. HR values from multivariate Cox regression analysis were used as preferred. Otherwise, data from univariate analysis will be used for analysis. (VI) Study type is a randomised controlled trial or prospective study or retrospective study.

Articles were excluded from the study if they met any of the following exclusion criteria: (I) concerned a review, animal cell experiment, or conference paper, and/or the full text of the article was not available; (II) had a sample size less than 20 and the 95% CIs could not be obtained.

### Data extraction and quality evaluation

The literature screening and data extraction were performed independently by two researchers. If a disagreement arose, consensus was reached via discussion. The following relevant information was extracted from the final included studies: first author, publication year, region, study type, treatment details, sample size, sex, age, time of ALBI acquisition, OS, PFS, objective response rate (ORR), disease control rate (DCR), and their related effect estimates. The ALBI grade was calculated based on the serum albumin and bilirubin levels. The following formula was used [32]: {ALBI score = [log10 bilirubin (µmol/L) × 0.66] + [albumin (g/L) ×  − 0.085]}. Classification standard: Grade 1, ALBI ≤  − 2.60; Grade 2, − 2.60 < ALBI ≤  − 1.39; Grade 3, ALBI >  − 1.39. The modified ALBI grade was calculated based on the values of ALBI as following: Grade 1, ALBI ≤  − 2.60; Grade 2a, − 2.60 < ALBI ≤  − 2.27; Grade 2b, − 2.27 < ALBI ≤  − 1.39; Grade 3, ALBI >  − 1.39.

The Newcastle–Ottawa Scale (NOS) was used to assess the quality of the included studies [33]. The assessment was done by Cao and Zhou, independently. All studies were evaluated on the basis of their cohort selection, comparability, and outcome measures. The maximum NOS score is 9, Studies scoring 0–3 were rated as low quality; 4–6 as moderate quality; and a study scoring 7–9 was rated as high quality [34, 35]. The main limitations that reduced the overall quality of the included studies were two: the decline in follow-up rates and the comparability of confounding factors.

### Statistical analysis

Review Manager 5.4 software was used for the statistical analysis [36]. The ORs/HRs and 95% CIs were obtained from univariate analysis of the included studies, and the OR/HR values were combined to calculate the combined estimates. A HR value > 1 indicated that high-grade ALBI was associated with a poor prognosis in HCC. The OR values and their 95% CIs were calculated for the dichotomous variables in the study.

Heterogeneity was assessed using the I^2^ statistic. An I^2^ value ≥ 50% and a *P* value < 0.1 indicated heterogeneity between the studies, and a random-effects model was used. A two-tailed *P* value < 0.05 indicated a statistical difference. I^2^ provides an estimate of the proportion of variance in the outcomes not attributable to sampling error alone, thus reflecting the actual heterogeneity in the outcomes. Funnel plots were directly observed to assess whether there was publication bias, and the asymmetric distribution of studies indicated publication bias. Sensitivity analyses were performed by including and excluding studies one by one. A *P* value < 0.05 indicated a statistically significant difference.

## Results

### Literature search results

**Fig. 1:**
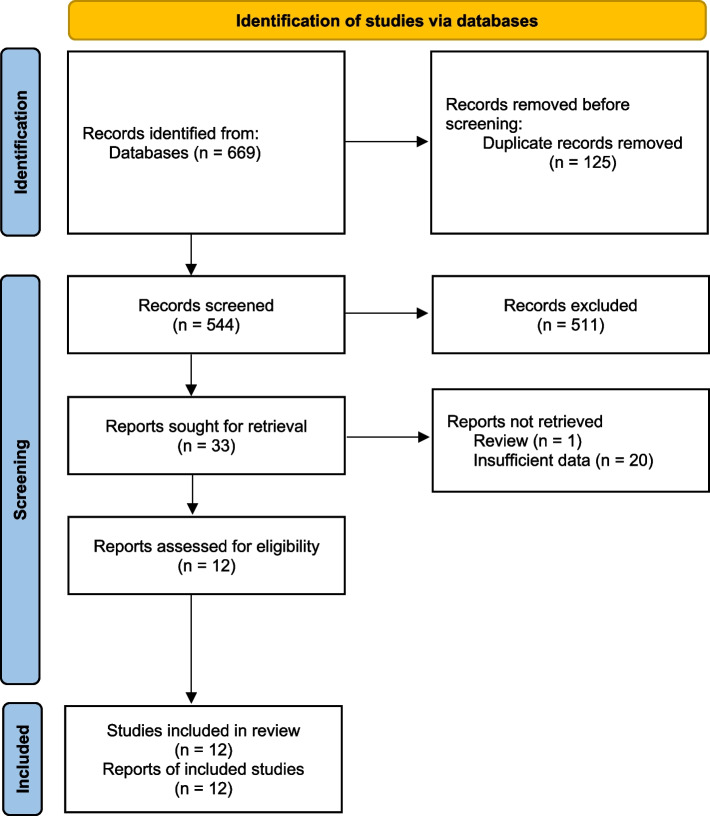
Flow chart showing the process used to select the eligible studies

A total of 669 articles were retrieved by searching the databases. After 125 duplicate articles had been removed, 511 additional articles were excluded after reading the titles and abstracts, and a further 21 articles were excluded after reading the full texts of the articles. Ultimately, 12 studies [30–32, 37–45] with 1,918 patients were included in the meta-analysis (Fig. 1). The NOS scores of the included studies were all ≥ 7, indicating that the included studies were of good quality (Table 1).

**Table 1:** The quality assessment results by NOS criteria

Author	Year	Selection				Comparability	Outcome			Score
		A	B	C	D	E	F	G	H	
Hitomi Takada	2019	☆	☆	☆	–	☆☆	☆	☆	–	7
Atsushi Yukimoto	2019	☆	☆	☆	–	☆☆	☆	☆	–	7
Hyung-Don Kim	2020	☆	☆	☆	☆	☆☆	☆	☆	☆	9
Satoshi Komiyama	2020	☆	☆	☆	☆	☆☆	☆	☆	–	8
Yeonghak Bang	2021	☆	☆	☆	☆	☆☆	☆	☆	☆	9
Young Mi Hong	2021	☆	☆	☆	☆	☆☆	☆	☆	☆	9
Hironori Ochi	2021	☆	☆	☆	☆	☆☆	☆	☆	☆	9
Margherita Rimini	2021	☆	☆	☆	☆	☆☆	☆	☆	☆	9
Takayuki Tokunaga	2021	☆	☆	☆	☆	☆☆	☆	☆	☆	9
Hung-Wei Wang	2021	☆	☆	☆	☆	☆☆	☆	☆	☆	9
Po-Yao Hsu	2022	☆	☆	☆	☆	☆☆	☆	☆	☆	9
I-Cheng Lee	2022	☆	☆	☆	☆	☆☆	☆	☆	☆	9

“Selection” part includes A: representativeness of cases, B: selection of controls, C: exposure ascertainment, and D: no death when investigation begin. “Comparability” part includes E: comparable on confounders. “Outcome” part includes F: outcome assessment, G: adequate follow-up, and H: loss to follow-up rate. The total score is equal to the total number of stars

*NOS* Newcastle–Ottawa Scale

### Characteristics of the included studies

**Fig. 2:**
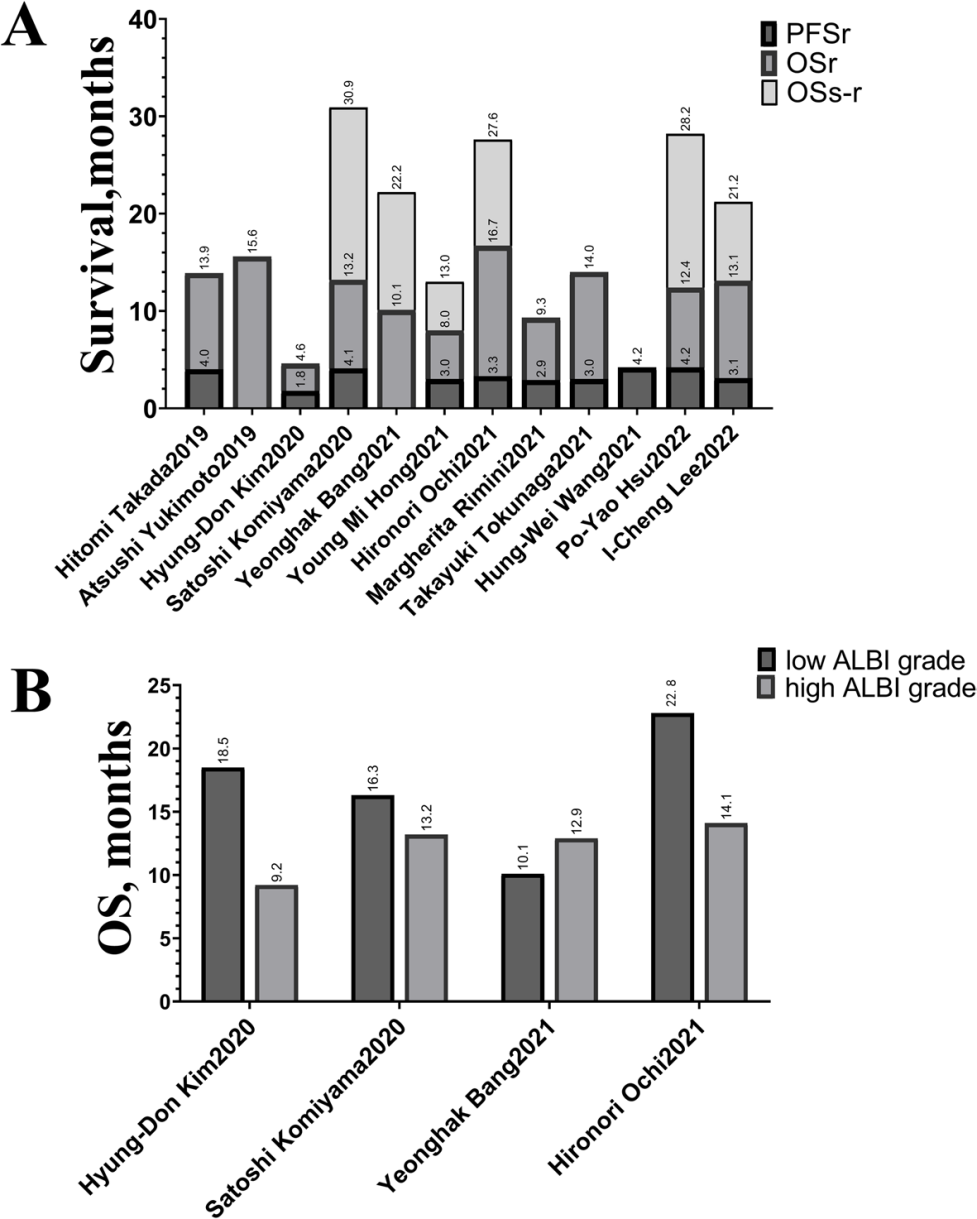
The pooled individual OS and PFS from the included studies. **A** PFS and OS from the initiation of regorafenib, and OS from the start of sorafenib to regorafenib. **B** Individual OS for low-grade ALBI *vs.* high-grade ALBI. OS, overall survival; PFS, progression-free survival; ALBI, Albumin-Bilirubin

A total of 12 retrospective studies [30–32, 37–45] were included in this meta-analysis, of which 3 were from China, 7 were from Japan, and 2 were from Europe. Among the 12 studies, 3 investigated the feasibility of the ALBI grade in assessing the acceptability of regorafenib, 11 investigated OS, and 10 investigated the PFS outcomes (Fig. 2). All the patients received first-line sorafenib therapy, and in 10 articles, the patients received subsequent regorafenib therapy (Table 2).

**Table 2:** Baseline characteristics of the included studies

Author	No	Region	Sex (male)	Age	Cancer type	BCLC B	BCLC C	ALBI grade			Outcomes
								1	2	3	
Hitomi Takada, 2019	190	Asia	149 (78%)	72	unresectable HCC	86	104	50	140	0	Predictors, OS, efficacy
Atsushi Yukimoto, 2019	138	Asia	122 (88%)	68	sorafenib-treated HCC	41	97	NR	NR	NR	Predictors, OS
Hyung-Don Kim, 2020	499	Asia	50 (85%)	58	Child–Pugh B HCC	18	481	188	288	23	Predictors, OS, efficacy
Satoshi Komiyama, 2020	21	Asia	18 (86%)	72	advanced HCC	10	11	5	16	0	Predictors, OS, PFS, efficacy
Yeonghak Bang, 2021	198	Europe	174 (88%)	NR	sorafenib-progressed and -tolerated HCC	20	178	186	12	0	Predictors, OS, efficacy
Young Mi Hong, 2021	58	Asia	53 (91%)	60	sorafenib-treated HCC	15	43	26	32	0	Predictors, OS, efficacy
Hironori Ochi, 2021	51	Asia	39 (74%)	71	unresectable HCC	17	31	14	37	0	Predictors, OS, PFS, efficacy
Margherita Rimini, 2021	284	Europe	253 (89%)	68	advanced HCC	20	264	NR	NR	NR	Predictors, OS, efficacy
Takayuki Tokunaga, 2021	197	Asia	161 (82%)	NR	sorafenib-treated HCC	64	127	41	55	NR	Predictors, OS, efficacy
Hung-Wei Wang, 2021	88	Asia	69 (78%)	65	unresectable HCC	23	64	54	33	0	Predictors, OS, PFS, efficacy
Po-Yao Hsu, 2022	86	Asia	66 (77%)	66	unresectable HCC	12	74	65	21	0	Predictors, OS, PFS, efficacy
I-Cheng Lee, 2022	108	Asia	91 (84%)	65	unresectable HCC	20	88	44	63	1	Predictors, OS, PFS, efficacy

*BCLC* Barcelona clinic liver cancer, *ALBI* Albumin-Bilirubin, *HCC* Hepatocellular carcinoma, *NR* Not reported, *OS* Overall survival; *PFS* Progression-free survival

### Meta-analysis results

#### The relationship between the baseline ALBI grade and eligibility for regorafenib therapy

**Fig. 3:**
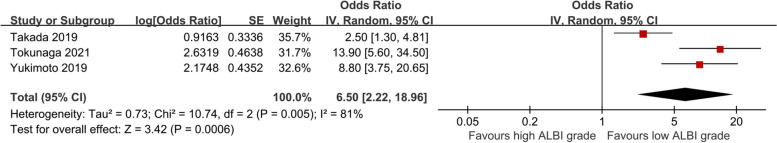
The forest plots of ALBI grade application in determining the eligibility of patients for regorafenib treatment. SE, standard error; CI, confidence interval; ALBI, Albumin-Bilirubin; ORR, objective response rate; DCR, disease control rate

Among the 12 studies, 3 analyzed whether ALBI grade could predict which patients would be candidates for regorafenib therapy [32, 37, 42]. The results of the meta-analysis showed that there was significant heterogeneity among these studies (I^2^ = 81%, *P* = 0.005), and thus a random-effects model was used (Fig. 3). HCC patients with low ALBI grades were more suitable for subsequent regorafenib therapy than those with high ALBI grades (OR = 6.50, 95% CI: 2.22–18.96, *P* < 0.01).

#### The relationship between the baseline ALBI grade and the efficacy of regorafenib in HCC patients

The meta-analysis was not performed due to limited number of included studies. Therefore, the descriptive analysis was used in this analysis. Overall, by combining the individual data, regorafenib treatment [38, 43] was 1.98 times more effective in HCC patients with low-grade ALBI at the baseline than in patients with high-grade ALBI at the baseline (OR = 1.98, *P* = 0.19). The DCR [38, 43] of HCC patients with baseline low-grade ALBI was 2.90 times higher than that of patients with baseline high-grade ALBI (OR = 2.90, *P* = 0.003).

#### The relationship between the baseline ALBI grade and OS after regorafenib treatment in HCC patients

**Fig. 4:**
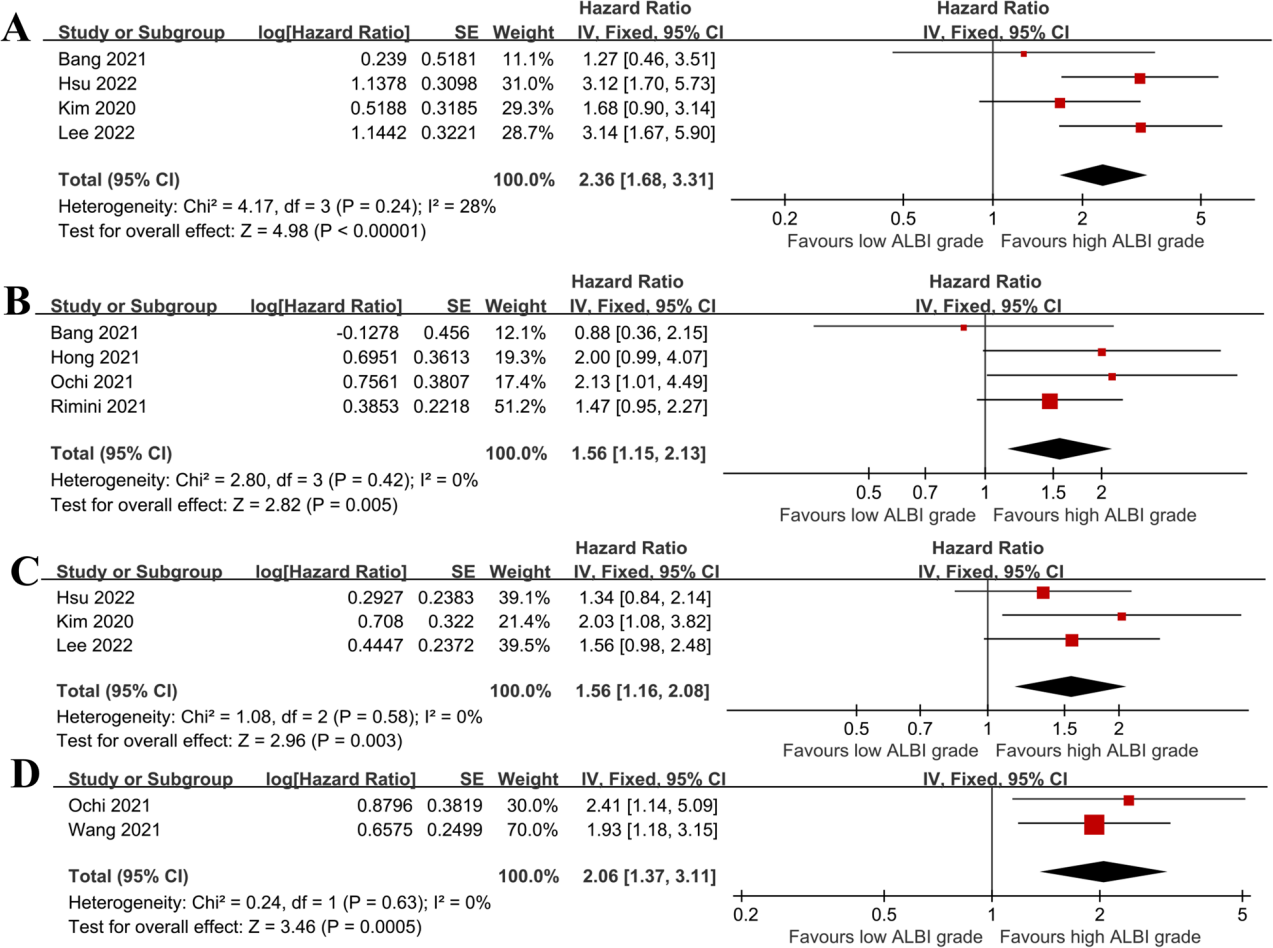
The forest plots of OS and PFS in the regorafenib-treated HCC patients with different grades of ALBI. **A** OS benefit determined by the baseline ALBI grade before sorafenib treatment; **B** OS results determined by the baseline ALBI grade before regorafenib treatment; **C** PFS benefit determined by the baseline ALBI grade before sorafenib treatment; **D** PFS results derived from baseline ALBI before regorafenib treatment. SE, standard error; CI, confidence interval; OS, overall survival; PFS, progression-free survival; HCC, hepatocellular carcinoma; ALBI, Albumin-bilirubin

A total of 7 studies [30, 31, 38, 40, 41, 44, 45] examined the relationship between the baseline ALBI grade and OS. We performed subgroup analyses based on the baseline ALBI definitions. First, the relationship between the ALBI grade and OS was analyzed using the baseline ALBI grade before sorafenib treatment. As no significant heterogeneity was found, a fixed-effects model was used for the analysis (Fig. 4A). The results of the meta-analysis showed that the mortality risk of HCC patients with high-grade ALBI was 2.36 times that of patients with low-grade ALBI (HR = 2.36, 95% CI: 1.68–3.31, *P* < 0.00001). No significant heterogeneity was found in relation to the baseline ALBI grade before regorafenib treatment, and thus a fixed-effects model was used (Fig. 4B). The meta-analysis results showed that high-grade ALBI was significantly associated with a higher risk of death than low-grade ALBI (HR = 1.56, 95% CI: 1.15–2.13, *P* = 0.005).

As modified ALBI was the further extension of ALBI grade, we also examined the prognostic role of the modified ALBI grade. Among the studies, 3 reported on the association between the modified ALBI grade and the survival of HCC patients after regorafenib treatment [30, 39, 41]. Komiyama et al*.* performed an analysis of OS based on the modified ALBI grade, and found that patients with 1 or 2a grade ALBI had a median OS of 16.3 months, while those with grade 2b had a median OS of 13.2 months (*P*= 0.96) [39]. Rimini et al*.* reported that HCC patients with grade 2a (HR = 1.63, *P* > 0.05) or 2b (HR = 1.30, *P*> 0.05) ALBI had a worse OS than those with grade 1 ALBI [30]. However, compared to patients with grade 1 or 2a, those with grade 2b or 3 ALBI had a significantly worse OS (HR = 2.13, *P*= 0.047) [41].

#### Relationship between the baseline ALBI grade and PFS in patients with HCC treated with regorafenib

A total of 4 studies [38, 41, 43, 44] reported on the relationship between the baseline ALBI grade and PFS. The relationship between the ALBI grade and PFS was analyzed using the baseline ALBI grade before sorafenib treatment. As no significant heterogeneity was found, a fixed-effects model was used for this analysis (I^2^ = 0%, *P* = 0.58). The meta-analysis (Fig. 4C) showed that HCC patients with high-grade ALBI had a 1.56-fold higher risk of disease progression than those with low-grade ALBI (HR = 1.56, 95% CI: 1.16–2.08, *P* = 0.003). In relation to the baseline ALBI grade before regorafenib treatment, as no significant heterogeneity was found, a fixed-effects model was used (I^2^ = 0%, *P* = 0.63). The meta-analysis results (Fig. 4D) showed that patients with high-grade ALBI had a significantly higher risk of disease progression than those with low-grade ALBI (HR = 2.06, 95% CI: 1.37–3.11, *P* = 0.0005).

### Publication bias and sensitivity analysis

The funnel plots are presented in Fig. S1. Data for OS and PFS were used to assess the publication bias of the included studies, and the funnel plots showed that the included studies were basically symmetrical on both sides of the funnel, suggesting that the risk of publication bias was low. To further assess the bias, we calculated the Egger’s and Begg’s tests, and the results revealed that there was no significant publication bias in relation to the association between the ALBI grade and OS (Egger’s test = 0.95, Begg’s test = 1.00), but not for the ALBI grade and PFS (Egger’s test = 0.03, Begg’s test = 0.30).

Sensitivity analyses were conducted to evaluate the potential effect of each included study on the summarized data. From the analyses, the pooled estimates were not significantly altered after excluding each individual study in turn (Fig. S2).

## Discussion

In recent years, following its wide clinical application, regorafenib has been shown to have good efficacy and tolerable safety in the treatment of HCC; however, few studies have focused the prognostic biomarkers of regorafenib [46, 47]. The present study showed that baseline high-grade ALBI in HCC patients is associated with poor OS and PFS. Further, the subgroup analysis showed that the baseline ALBI grade both before sorafenib or regorafenib treatment predicted the efficacy of regorafenib and the prognosis of HCC patients. There was also a significant difference in the DCR between the high-grade ALBI group and the low-grade ALBI group (both *P* < 0.05). Further, the ALBI grade can also be used to assist in determining whether HCC patients are suitable for regorafenib treatment.

The ALBI grade is a novel liver function classification system, compared to the Child–Pugh classification system. The ALBI grade has been shown to have good prognostic value in recent studies [48, 49]. In the prognostic prediction analysis of patients treated with TACE and sorafenib, the ALBI grade was found to be superior to the Child–Pugh grade. In a systematic review, the investigators compared the prognostic predictive value of the ALBI grade and Child–Pugh grade in patients with HCC, and found that ALBI grade had better discriminative ability than Child–Pugh grade in the prognosis of HCC patients [48]. The prognostic value of the ALBI grade and Child–Pugh grade was also evaluated in patients with HCC treated with TACE. In a study cohort of 303 patients [49], the survival curves stratified by the ALBI grade were significantly distinct (*P*< 0.001), which suggested that the ALBI grade had greater prognostic value than the Child–Pugh grading for HCC patients receiving TACE. Another study showed that the combined application of the ALBI and Child–Pugh grades reduced the heterogeneity of liver cancer patients with different liver function states [38]. In a study by Kim et al*.*, only patients with BCLC stage B HCC were included. After regorafenib treatment, the median PFS and OS of the ALBI grade 1/2 and ALBI 3 patients were 2.37 *vs.* 1.08 months, and 5.55 *vs.*3.64 months, respectively [38]. Takada et al [37]. noted that according to the Child–Pugh classification, patients with a Child–Pugh score of 5 had longer OS than those with a Child–Pugh score of 6 (18.6 *vs.* 7.9 months, *P* < 0.01). In relation to the ALBI grade, patients with ALBI grade 1 had longer OS than those with ALBI grade 2 (21.6 *vs.* 9.3 months, *P* = 0.001). Taken together, these results suggest that patients with good liver function reserve achieve better survival after receiving regorafenib treatment.

Current, evidence suggests that the ALBI grade has predictive value in a variety of tumors, including HCC. Marasco et al*.*evaluated the diagnostic value of the ALBI grade in predicting post-hepatectomy liver failure in HCC patients who underwent liver resection [50]. In their meta-analyses, a total of 7 studies were included, and the results showed that patients with high-grade ALBI at the baseline had increased rates of liver failure compared to those with low-grade ALBI at the baseline [50]. Another meta-analysis also evaluated the prognostic role of the baseline ALBI grade in HCC patients after surgical resection [51]. That meta-analysis included 20 studies with 11,365 patients, and the combined results showed that high-grade ALBI was correlated with short OS (HR = 1.64, *P* < 0.01) and PFS (HR = 1.42, *P*< 0.01) [51]. In addition, this correlation was not significantly influenced by region or sample size. The clinical utility of ALBI as a prognostic factor was also assessed in HCC patients undergoing TACE [52]. By extracting data from 8 studies, the authors found that a higher ALBI grade at the baseline was associated with a poor prognosis, and a median OS of 12.0 months in ALBI grade 3 compared to those with a median OS of 33.5 months in ALBI grade 1 [52]. Recently, changes in ALBI score have been found to be a prognostic factor in patients receiving atezolizumab and bevacizumab (Atez/Bev). Unome et al*.*, in 62 unresectable HCC patients treated with Atez/Bev, ALBI scores deteriorated significantly within 3 months and were an independent prognostic factor for treatment [53]. Campani et al*.* enrolled 58 HCC patients treated with Atez/Bev, the results showed that the combination of ALBI and AFP early response was significantly associated with poor prognosis in patients with ALBI2-AFP non-responsive patients with OS (*P* = 0.046) and PFS (*P*= 0.012). These results indicate that ALBI grading combined with early AFP response can improve prognostic differentiation [54]. In addition, a study using artificial intelligence algorithms to study serum biomarkers and construct survival prediction models, which show that ALBI grading is one of the most important serum biomarkers related to OS [55]. All the above studies suggest that the baseline ALBI grade has an effective prognostic role in predicting prognosis, and these findings support our results.

However, the clinical value of the ALBI grade in predicting the prognosis of HCC patients treated with regorafenib is not well established. In our study, we assessed the association between the ALBI grade and prognosis of HCC patients who had undergone regorafenib treatment, and found that the baseline ALBI grade was significantly correlated with the survival and disease control outcomes, and its prognostic value was not affected by the definition of the baseline ALBI grade. High-grade ALBI was associated with a poor baseline ALBI prognosis in this population. Additionally, the ALBI grade could also be used to predict which HCC patients would be suitable for regorafenib treatment. Further, the modified ALBI grade was also suggested to be associated with survival outcomes, despite its prognostic value not being significant in the reported studies. The above findings suggest that the prognostic role of the ALBI grade may be applied in clinical practice. Our findings are consistent with the above studies, indicating that the ALBI grade has a certain generality and utility in efficacy prediction.

This study had some limitations. First, as the included studies were all retrospective studies, the baseline data may be inconsistent, and the data may be biased. Second, the included studies differed in terms of the treatment and follow-up, which may increase the heterogeneity of the analysis results. Subgroups, meta-regression and network meta-analysis should be performed. However, we failed to conduct them due to insufficient number of studies, comparisons, and sample sizes. Third, different time points for the ALBI data acquisition may have affected the predictive value of ALBI. Fourth, as some of the included studies only provided PFS data or only survival curves, this limited the studies that could be included in the meta-analysis, which in turn affected the predictive value of the ALBI grade in terms of OS. In addition, since the change in the ALBI score may be prognostic in patients receiving atezolizumab and bevacizumab, it should be studied the clinical value of the ALBI grade in patients that receive tyrosine kinase (TK) inhibitors after novel first-line combinations for HCC including immunotherapies. Therefore, large prospective multicenter study should be conducted to further explore its prognostic value.

## Conclusions

The ALBI grade is a simple and objective method for assessing liver function in HCC patients. It may predict the prognosis of HCC patients treated with regorafenib and could be used to help determine whether patients are suitable for regorafenib treatment, especially for those who are ALBI grade 1. In the meantime, the application of machine learning intelligence based on serum biomarker dosing may constitutes a springboard to reshape future research.

### Supplementary Information

**Additional file 1:** **Figure S1 **Funnel plots of HR OS determined by the baseline ALBI grade before sorafenib treatment (A), HR OS determined by the baseline ALBI grade before regorafenib treatment (B), HR PFS determined by the baseline ALBI grade before sorafenib treatment (C), and HR PFS determined by the baseline ALBI grade before regorafenib treatment (D). HR, hazard ratio; OS, overall survival; ALBI, albumin-bilirubin; PFS, progression-free survival; SE, standard error.

**Additional file 2:** **Figure S2 **Sensitivity analysis of HR OS determined by the baseline ALBI grade before sorafenib treatment (A), HR OS determined by the baseline ALBI grade before regorafenib treatment (B), HR PFS determined by the baseline ALBI grade before sorafenib treatment (C), and HR PFS determined by the baseline ALBI grade before regorafenib treatment (D). HR, hazard ratio; OS, overall survival; ALBI, albumin-bilirubin; CI, confidence interval.

## Data Availability

The datasets used and/or analysed during the current study available from the corresponding author on reasonable request.

## Abbreviations

ALBI: Albumin-Bilirubin
BCLC: Barcelona Clinic Liver Cancer
CI: Confidence interval
DCR: Disease control rate
HCC: Hepatocellular carcinoma
HR: Hazard ratio
NOS: Newcastle-Ottawa Scale
OR: Odds ratio
ORR: Response rate
OS: Overall survival
PFS: Progression-free survival
TACE: Transarterial chemoembolization
TK: Tyrosine kinase
