# Supplementary material for: Baseline Albumin-Bilirubin grade as a predictor of response and outcome of regorafenib therapy in patients with hepatocellular carcinoma: a systematic review and meta-analysis
Source: BMC Cancer. 2023 Oct 19;23:1006. doi: 10.1186/s12885-023-11488-9 (PMC10588229; doi:10.1186/s12885-023-11488-9)
Supplement: Supplementary file 1 — Additional file 1: Figure S1 Funnel plots of HR OS determined by the baseline ALBI grade before sorafenib treatment (A), HR OS determined by the baseline ALBI grade before regorafenib treatment (B), HR PFS determined by the baseline ALBI grade before sorafenib treatment (C), and HR PFS determined by the baseline ALBI grade before regorafenib treatment (D). HR, hazard ratio; OS, overall survival; ALBI, albumin-bilirubin; PFS, progression-free survival; SE, standard error. [file 12885_2023_11488_MOESM1_ESM.pdf]

A

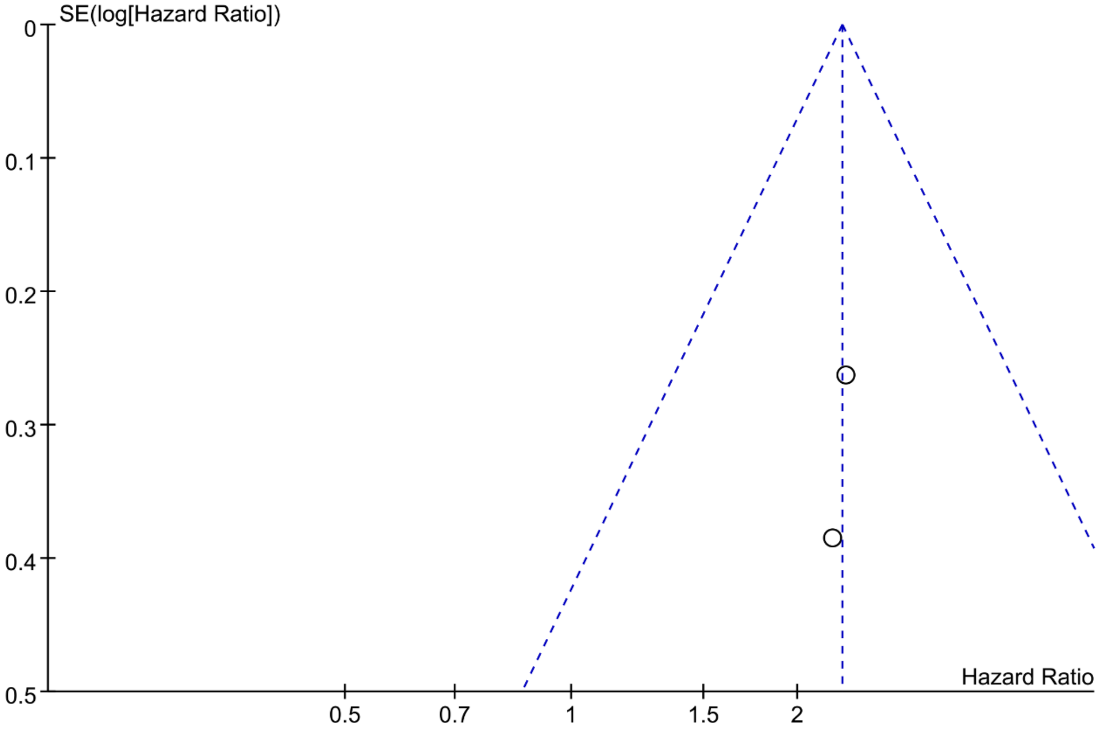

SE(log[Hazard Ratio])
0
0.1
0.2
0.3
0.4
0.5
Hazard Ratio
0.5
0.7
1
1.5
2

B

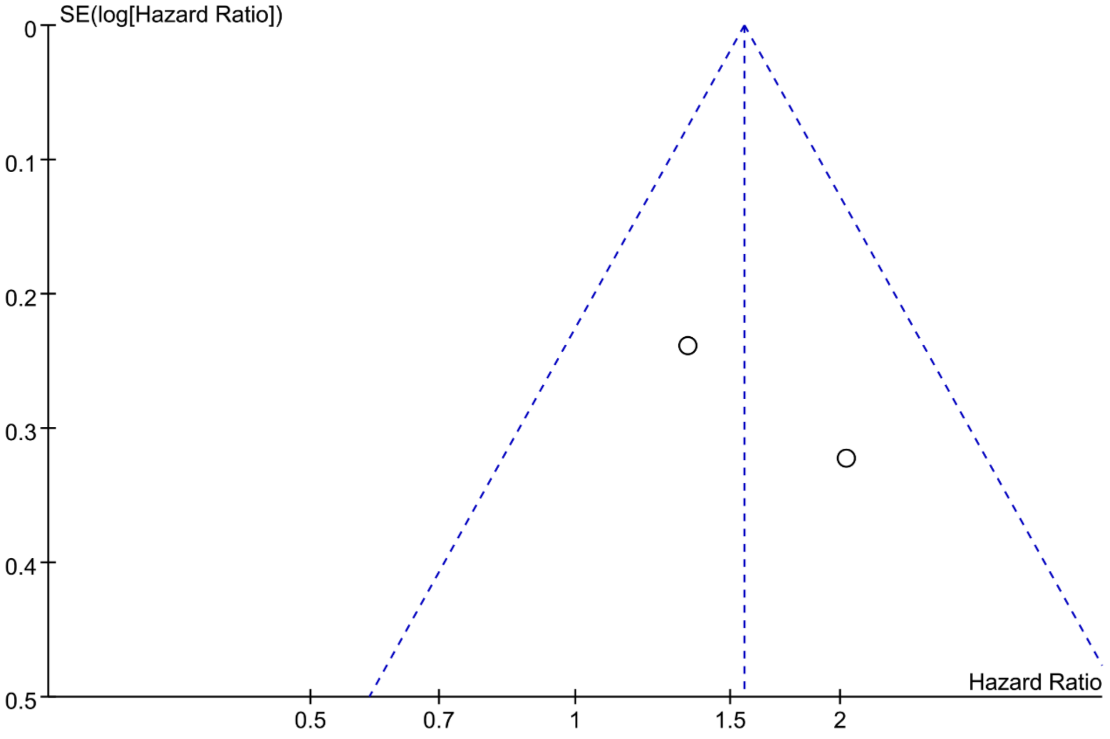

SE(log[Hazard Ratio])
0
0.1
0.2
0.3
0.4
0.5
Hazard Ratio
0.5
0.7
1
1.5
2

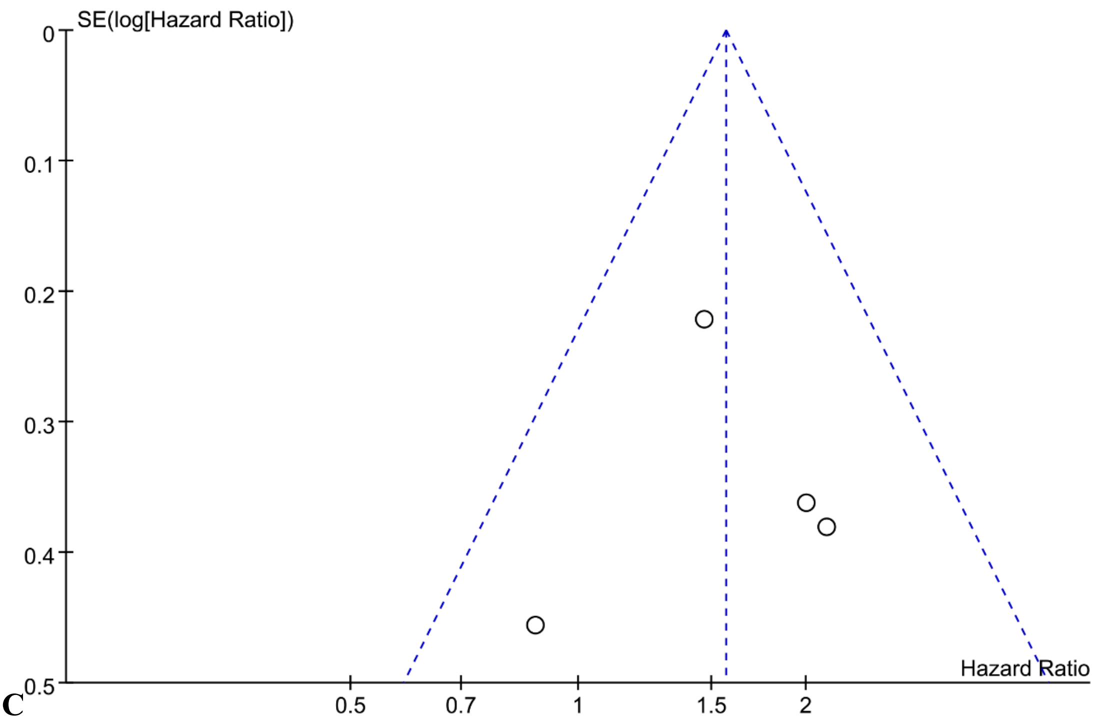

C

D

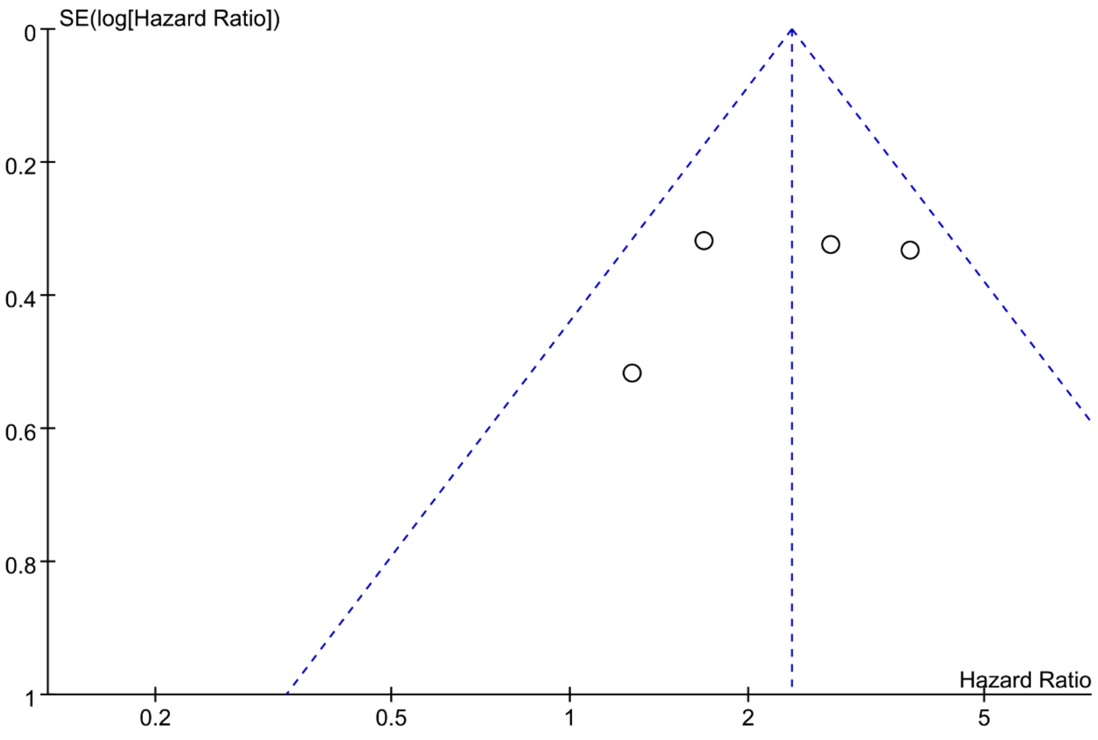

Figure S1 Funnel plots of HR OS determined by the baseline ALBI grade before sorafenib treatment (A), HR OS determined by the baseline ALBI grade before regorafenib treatment (B), HR PFS determined by the baseline ALBI grade before sorafenib treatment (C), and HR PFS determined by the baseline ALBI grade before regorafenib treatment (D). HR, hazard ratio; OS, overall survival; ALBI, albumin-bilirubin; PFS, progression-free survival; SE, standard error.
