# Supplementary material for: Baseline Albumin-Bilirubin grade as a predictor of response and outcome of regorafenib therapy in patients with hepatocellular carcinoma: a systematic review and meta-analysis
Source: BMC Cancer. 2023 Oct 19;23:1006. doi: 10.1186/s12885-023-11488-9 (PMC10588229; doi:10.1186/s12885-023-11488-9)
Supplement: Supplementary file 2 — Additional file 2: Figure S2 Sensitivity analysis of HR OS determined by the baseline ALBI grade before sorafenib treatment (A), HR OS determined by the baseline ALBI grade before regorafenib treatment (B), HR PFS determined by the baseline ALBI grade before sorafenib treatment (C), and HR PFS determined by the baseline ALBI grade before regorafenib treatment (D). HR, hazard ratio; OS, overall survival; ALBI, albumin-bilirubin; CI, confidence interval. [file 12885_2023_11488_MOESM2_ESM.pdf]

A

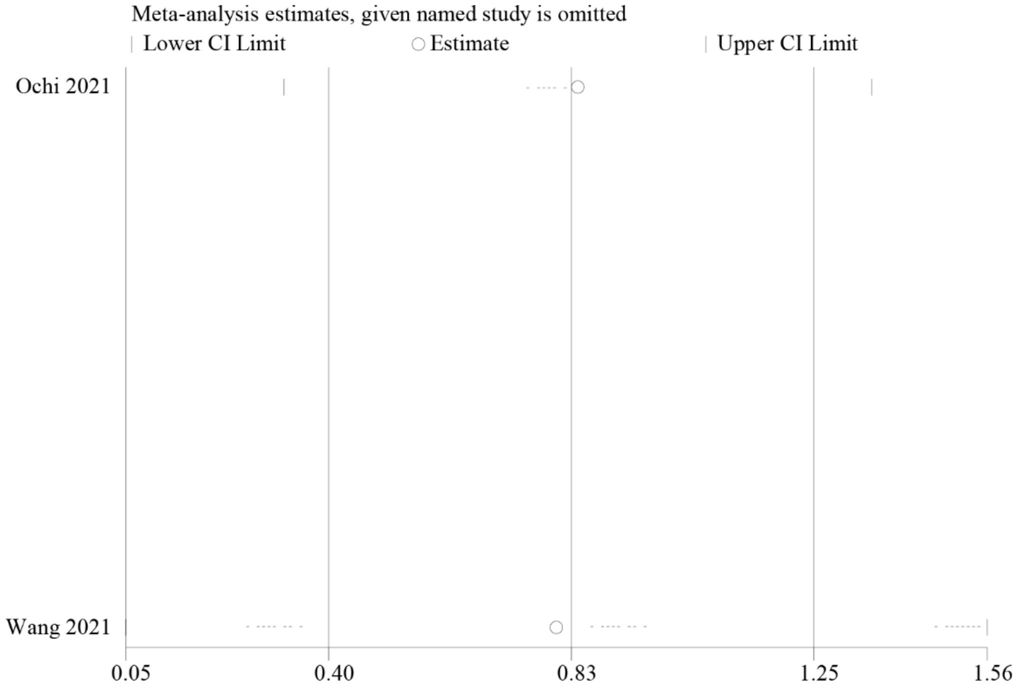
Meta-analysis estimates, given named study is omitted
Lower CI Limit
Estimate
Upper CI Limit
Ochi 2021
Wang 2021
0.05
0.40
0.83
1.25
1.56

B

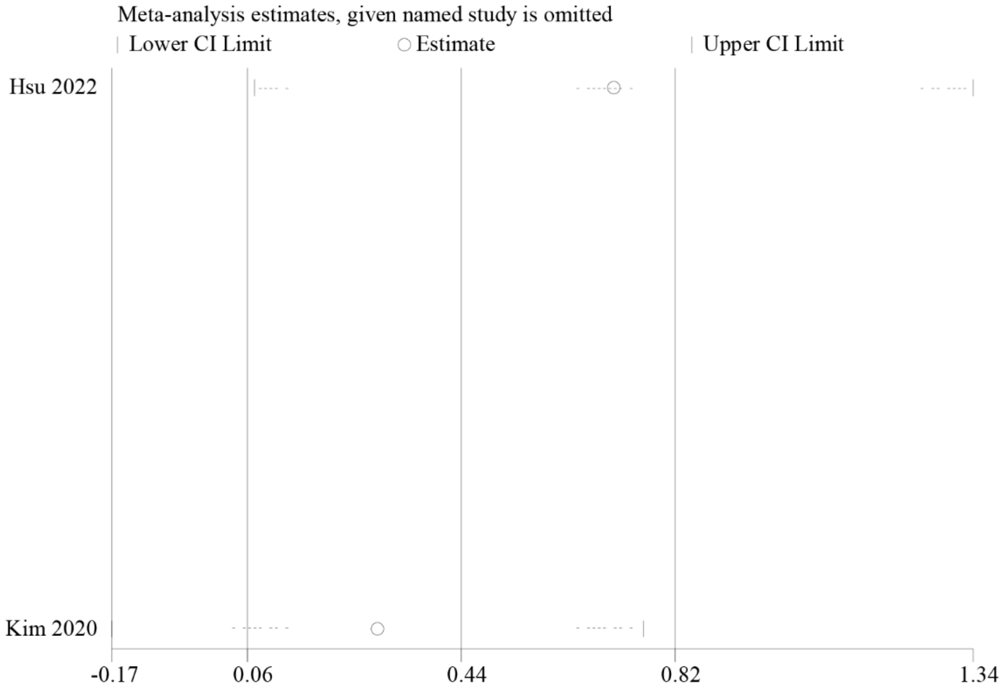
Meta-analysis estimates, given named study is omitted
Lower CI Limit
Estimate
Upper CI Limit
Hsu 2022
Kim 2020
-0.17
0.06
0.44
0.82
1.34

C

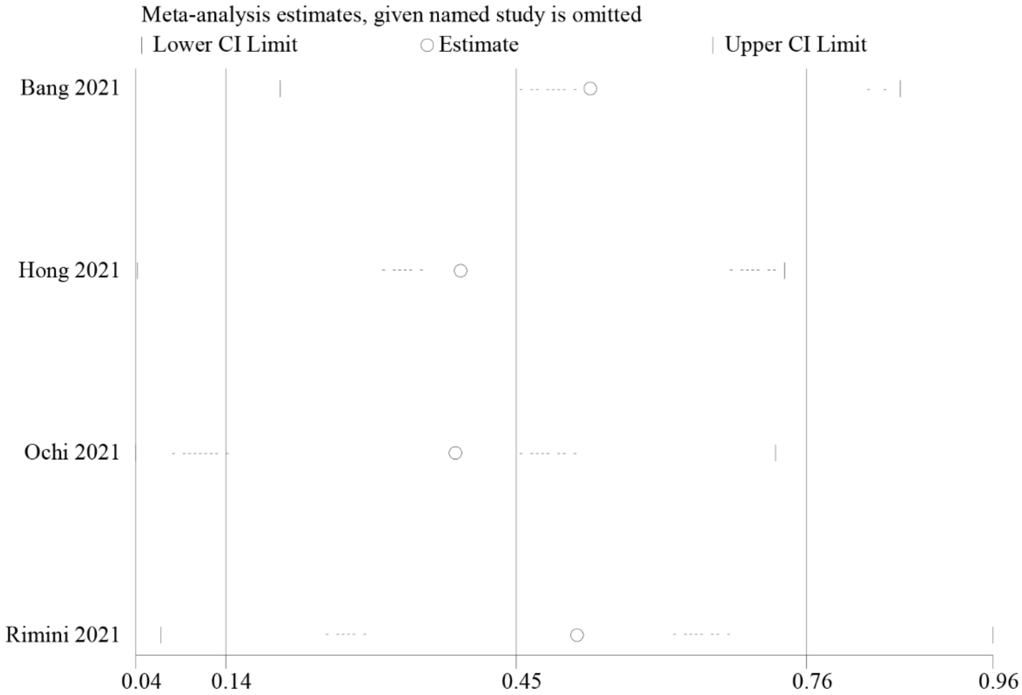

D

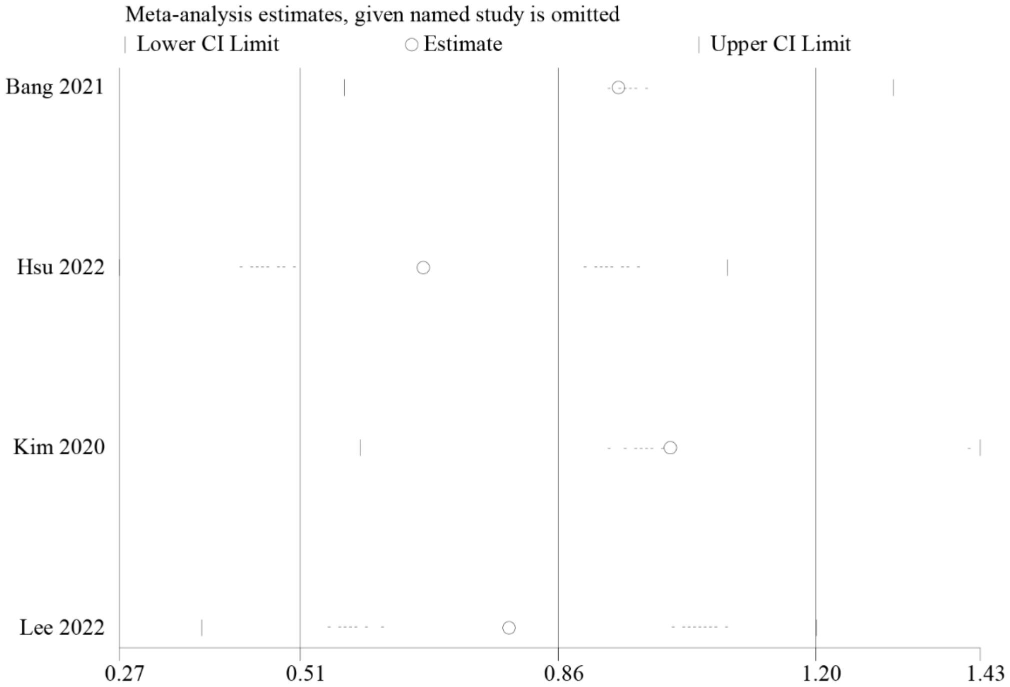

Figure S2 Sensitivity analysis of HR OS determined by the baseline ALBI grade before sorafenib treatment (A), HR OS determined by the baseline ALBI grade before regorafenib treatment (B), HR PFS determined by the baseline ALBI grade before sorafenib treatment (C), and HR PFS determined by the baseline ALBI grade before regorafenib treatment (D). HR, hazard ratio; OS, overall survival; ALBI, albumin-bilirubin; CI, confidence interval.
